# Supramolecular Polymer Brushes

**DOI:** 10.1021/acspolymersau.2c00067

**Published:** 2023-02-08

**Authors:** Friederike K. Metze, Harm-Anton Klok

**Affiliations:** Institut des Matériaux and Institut des Sciences et Ingénierie Chimiques, Laboratoire des Polymères, École Polytechnique Fédérale de Lausanne (EPFL), Bâtiment MXD, Station 12, CH-1015 Lausanne, Switzerland

**Keywords:** Polymer brushes, supramolecular chemistry, host−guest complexes, surface-initiated polymerization, grafting to, grafting from

## Abstract

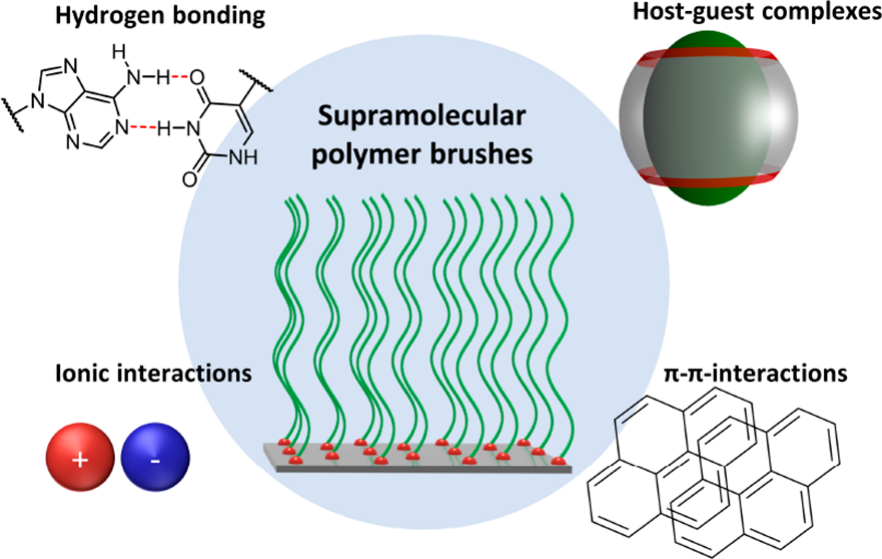

Polymer brushes are thin polymer films that consist of densely grafted, chain-end tethered polymers. These thin polymer films can be produced either by anchoring presynthesized chain-end functional polymers to the surface of interest (“grafting to”), or by using appropriately modified surfaces to facilitate growth of polymer chains from the substrate (“grafting from”). The vast majority of polymer brushes that have been prepared and studied so far involved chain-end tethered polymer assemblies that are anchored to the surface via covalent bonds. In contrast, the use of noncovalent interactions to prepare chain-end tethered polymer thin films is much less explored. Anchoring or growing polymer chains using noncovalent interactions results in supramolecular polymer brushes. Supramolecular polymer brushes may possess unique chain dynamics as opposed to their covalently tethered counterparts, which could provide avenues to, for example, renewable or (self-)healable surface coatings. This Perspective article provides an overview of the various approaches that have been used so far to prepare supramolecular polymer brushes. After presenting an overview of the various approaches that have been used to prepare supramolecular brushes via the “grafting to” strategy, examples will be presented of strategies that have been successfully applied to produce supramolecular polymer brushes via “grafting from” methods.

## 1 Polymer Brushes

Polymer brushes are assemblies of chain end-tethered polymer chains on solid substrates, which show remarkable properties as compared to their counterparts in solution, or polymer films obtained via, for example, drop- or spin-casting. Their nonbiofouling, ultralow friction, anticorrosive, colloidal stabilization, protein binding, adhesion, and wettability properties can be tailored by varying polymer composition, grafting density, film thickness, and polymer architecture.^1−5^ Polymer brushes have attracted interest not only for a range of biological applications that include artificial joints, drug delivery, antibiofouling surfaces, and biosensors but also for membrane and nanomaterials engineering.^2,3,6^

Two important molecular parameters that describe a polymer brush are the molecular weight of the polymer grafts (which is correlated with the dry film thickness) and the grafting density of the polymer brush. The grafting density σ is defined as the number of polymer chains per unit surface area, usually given as chains/nm^2^. With knowledge over the number-average molecular weight *M*_n_, the bulk polymer density ρ, and the dry film thickness *h*, the grafting density can be estimated with eq 1, where *N*_*A*_ is the Avogadro constant.^7^
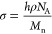
1

**Figure 1:**
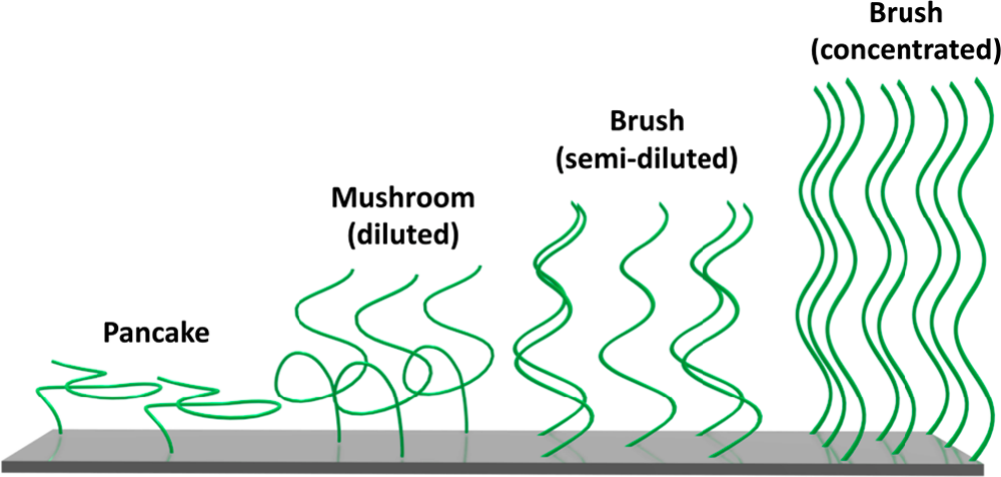
Different polymer brush conformations.

Depending on the grafting density, polymer molecular weight and possible polymer–substrate, and polymer solvent interactions, polymer brushes can adopt a variety of chain conformations, which are illustrated in Figure 1. A parameter to assess the conformation is the reduced grafting density ∑ (eq 2), which is a function of the grafting density σ and the radius of gyration of the polymer chains (*R*_g_).^8−11^ ∑ indicates the number of chains that occupy the surface area covered by a single chain under ideal conditions.^10^ Although the term “polymer brush” is often used to refer to any type of assembly of surface-tethered polymer chains, chain end grafted polymer thin films typically start to display the characteristic polymer brush scaling behavior for ∑ > 5.
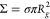
2

Decreasing the grafting density to ∑ ≤ 1, or lowering the molecular weight leads to collapse of the polymer chains, which then transition to the “mushroom” conformation. Also, polymer–substrate and polymer–solvent interactions can influence the conformation. If polymer–substrate interactions are strong, or polymer–solvent interactions are poor, a “pancake” formation is adapted (Figure 1).^1,3,11^

**Figure 2:**
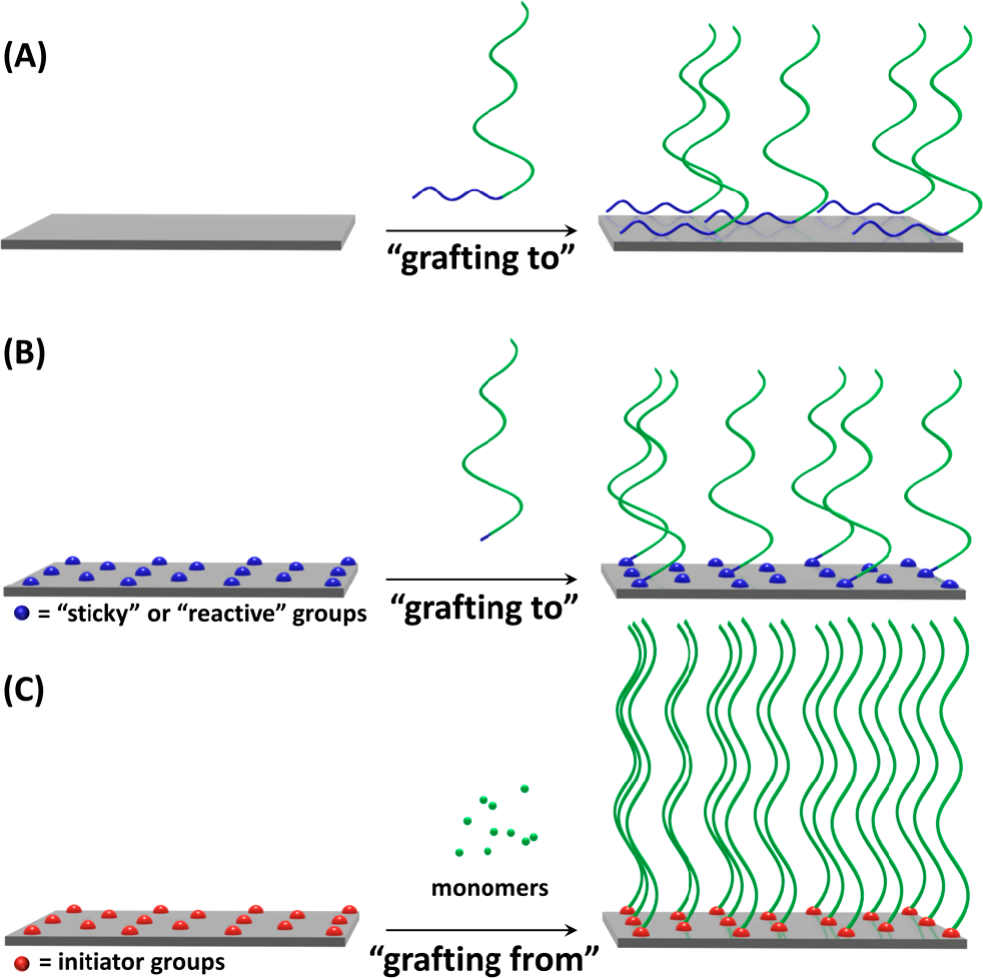
Polymer brushes prepared via “grafting to” using (A) the physisorption of block copolymers or (B) the chemical or physical adsorption of end-functionalized polymer chains and (C) the preparation of polymer brushes via the “grafting from” approach.

Since the properties of polymer brushes strongly depend on the conformation of the constituent polymer grafts, tuning the grafting density provides opportunities to engineer the properties of these polymer surfaces and interfaces. In general, there are two different methods to prepare polymer brushes, which are referred to as the “grafting from” and the “grafting to” methods. For the latter, two different possibilities exist: either the physisorption of block copolymers (Figure 2A) or the immobilization of end-functionalized polymer chains onto substrates that present complementary functional groups, which can allow for noncovalent (in case of “sticky” groups), or covalent attachment (when chemically reactive groups are presented) (Figure 2B).^3,8^ The “grafting to” method has the advantage that it uses presynthesized polymers, which can be fine-tuned regarding their composition, molecular weight, and architecture. However, the resulting polymer brush films typically have low grafting densities due to the excluded volume of the tethered polymer chains, and the limited diffusion of the macromolecules reaching the surface through the polymer layer. Higher grafting densities, extended polymer chain conformations, and thicker polymer layers, however, can be achieved with the “grafting from” method (Figure 2C). In this case, polymers are directly grown from surfaces that are modified with functional groups that can act as polymerization initiators or chain transfer agents.^3^ The use of controlled/living surface-initiated polymerization techniques for the synthesis of polymer brushes allows for a great level of control over polymer molecular weight, dispersity, architecture and functionality.^1,3^ The polymerization strategies that are most often used for the synthesis of polymer brushes via the grafting from method are controlled radial polymerization techniques such as reversible addition–fragmentation chain-transfer polymerization (RAFT),^12,13^ nitroxide-mediated radical polymerization (NMP),^14−16^ and atom transfer radical polymerization (ATRP).^17,18^

So far, polymer brushes are usually covalently anchored to the underlying surface. In contrast, the use of noncovalent interactions to chain-end tether polymers to a solid surface is much less explored. Anchoring or growing polymer chains using noncovalent interactions results in supramolecular polymer brushes. In the context of this article, supramolecular polymer brushes are defined as chain end-tethered assemblies of polymers grafted to a solid substrate via noncovalent interactions. Supramolecular brushes may possess unique chain dynamics as opposed to their covalently tethered counterparts, which could provide avenues to, for example, renewable or (self-)healable surface coatings. This perspective article will provide an overview of the various approaches that have been used so far to prepare supramolecular polymer brushes. In the remainder of this article, first the approaches that have been used to prepare supramolecular brushes via the “grafting to” strategy will be presented, followed by an overview of strategies that have been successfully applied to produce supramolecular polymer brushes via “grafting from” methods.

## 2 Supramolecular Polymer Brushes Prepared via “Grafting To”

The “grafting to” approach uses presynthesized polymers, which can be anchored to a solid substrate using a variety of noncovalent interactions. This section will give an overview of the approaches that have been used to prepare supramolecular polymer brushes via “grafting to” approaches, which include the physisorption of block copolymers, as well as the chain-end tethering via π–π interactions, electrostatic interactions, hydrogen bonds, and host–guest interactions.

### 2.1 Block Copolymer Physisorption

**Figure 3:**
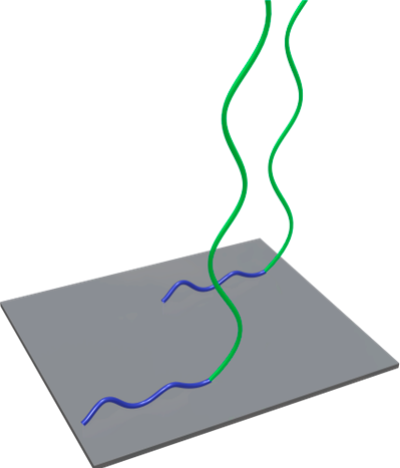
Supramolecular polymer brush prepared via block copolymer physisorption.

Block copolymer physisorption (Figure 3) does not produce supramolecular polymer brushes within the scope of the definition used in this article, since it does not result in purely chain end-anchored polymer assemblies. As much of the early work on block copolymer brushes, however, has paved the way to much of what is known now about polymer brushes, it seems appropriate to include this approach in this Perspective. The physisorption of block copolymers has been extensively used for the preparation of polymer brushes. Ideally, one of the blocks is anchored to the substrate (anchor block), while the other block stretches away from the surface (buoy block).^19−27^ Which block physisorbs can be controlled by the choice of the substrate or solvent, including its pH or ionic strength, making the process either “solvent selective” or “substrate selective”.^28,29^

One example of solvent-, or pH-selective brush formation via diblock copolymer adsorption was published by Sakai et al.^30^ In this study, silica surfaces were dip-coated into aqueous solutions of poly((2-diethylamino)ethyl methacrylate)-*b*-poly(methacrylic acid) (PDEA-*b*-PMAA) at pH 4 and 9 and characterized with a quartz crystal microbalance (QCM) as well as optical reflectometry (OR). When acidic solutions were used, the adsorbed copolymers form a flat layer on the surface, which is opposed to their core–shell micelle structure in solution. While the protonated PDEA block is adsorbed on the silica surface via electrostatic interactions, the hydrophobic PMAA block is likely to collapse at low pH. In alkaline environment, however, the PDEA blocks are adsorbed onto the surface while the deprotonated PMAA blocks stretch away from the surface, forming the polymer brush layer. Differences in the adsorbed mass obtained by QCM and OR revealed the degree of hydration. At pH 9, a significantly higher degree of hydration occurred as compared to pH 4, which further supports the hypothesis of the presence of polymer brushes that extend from the interface and adsorb a higher amount of fluid than the flat polymer layer at pH 4. However, after drying, the polymers aggregated to form micelle-like structures on the surfaces as shown with AFM measurements.

Poly(ethylene oxide)-*b*-polystyrene (PEO-*b*-PS) copolymers have also been used to form polymer brushes under solvent-selective as well as substrate-selective conditions. Munch and Gast investigated the adsorption of these block copolymers from cyclopentane, which is a good solvent for PS, but not PEO, on sapphire.^31^ From the observation that the block copolymer, but not the PS homopolymer, adsorbed from solution, it was concluded that a PS brush-like structure was formed. The adsorption process was 90% complete within 5 min of exposure to a flowing block copolymer solution and complete saturation was reached after 30 min. Washing with cyclopentane did not lead to desorption of the PEO blocks from the surface.

The adsorption of PEO-*b*-PS on mica or silica from a “non-selective” or good solvent for both blocks has been investigated in several studies, and it illustrates “substrate-selective” brush formation.^21,23,32,33^ Reasons for the selective adsorption of the PEO blocks are the higher affinity of the more polar block to the polar substrate and the repulsive forces between the polymer blocks. Another thoroughly investigated example of “substrate-selective” adsorption from “non-selective” solvents is the formation of polystyrene-*b*-poly(4-vinylpyridine) (PS-*b*-P4VP)^34,35^ and polystyrene-*b*-poly(2-vinylpyridine) (PS-*b*-P2VP)^36^ brushes on silica or mica from toluene or tetrahydrofuran (THF). Bazuin and co-workers, for example, deposited PS-*b*-P4VP on silicon surfaces by dip-coating from tetrahydrofuran or toluene.^35^ While the P4VP block adsorbs on the silicon surface through hydrogen bonding, the PS block formed the brush layer. Dry film thicknesses were measured with ellipsometry and were found to be concentration dependent. A film thickness of up to 18 nm was measured for the highest polymer concentration of 5 mg/mL. However, at these high concentrations, AFM experiments revealed the adsorption of additional material and the formation of micelle-like structures. Only for low polymer concentrations of up to 0.1 mg/mL, a perfect brush monolayer with a film thickness of 4 nm was observed. The presence of a dense layer of polymer brushes was further supported by the homogeneous deposition of gold nanoparticles (AuNP) on the polymer layer prepared at low concentrations. At high concentrations, however, when the micelle-like structure is favored, adsorbed AuNPs appeared in cluster-like structures. In many cases, brush formation competes with adsorption of micelle-like structures and changes of the polymer composition, polymer architecture, the substrate, or the solvent may favor the one over the other. Zdyrko et al., for example, found that the conformation of adsorbed poly(dimethylsiloxane) (PDMS) and poly(ethylene glycol) (PEG) copolymers of different composition and architecture depends on the PEG percentage, and is substrate-selective.^37^ All copolymer compositions formed micelle-like structures on the hydrophilic silicon surfaces. However, on the more hydrophobic hexadecane (C16) substrates, brush formation was observed for copolymers with higher PEG percentages, with the hydrophobic PDMS block bound to the surface.

The stability of adsorbed block copolymers has been investigated in several studies. One possibility to remove the polymer chains is exposure to a good solvent. When investigating the stability of PS-*b*-PEO brushes in toluene as a good solvent at different shear rates, surprisingly, the rate of desorption did not increase linearly with the shear rate. The polymer brushes were stable up until very high shear rates, and a rapid increase in desorption rate was measured only after a certain shear threshold was reached.^38,39^ Physisorbed block copolymers have also been found to be sensitive to the displacement by other polymers, low molecular weight compounds, and temperature.^28^ Efforts have been made to increase the stability of such surfaces. Poly(l-lactide)-*b*-poly(ethylene oxide) (PLLA-*b*-PEO) brushes on PLLA nanoparticles, with the PLLA block attached to the surface, were prepared by Chánova et al. via spin coating from acetone/methanol mixtures.^40^ The surfaces were not stable in aqueous solution after storage for several days. However, exposing to increased temperatures of 50 °C with subsequent quenching in cold water increased the phase separation and the stability of the surfaces by “kinetically freezing” the PLLA block. They also observed that using semicrystalline PLLA increased the stability as compared to amorphous PLLA.

### 2.2 π–π Interactions

**Figure 4:**
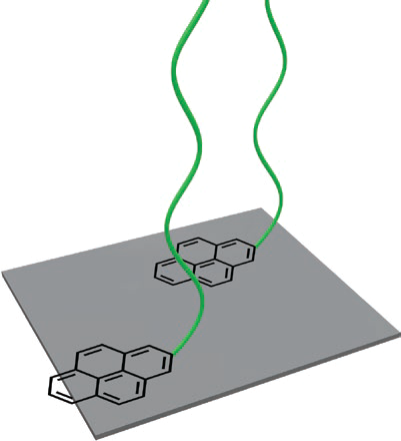
Supramolecular polymer brush tethered via π–π interactions.

The polyaromatic, planar structure of graphene allows small aromatic molecules to interact with the material via π–π-interactions (Figure 4). The noncovalent modification of graphene is of interest due to its conducting properties that derive from the sp^2^ hybridized carbon atoms.^41^

Pyrene-terminated poly(*N*-isopropylacrylamide) (PNIPAAm), synthesized via RAFT polymerization, was used to prepare PNIPAAm-modified temperature-responsive graphene sheets, since PNIPAAm collapses on the surface at temperatures above its lower critical solution temperature. The presence of the polymer was proven with Raman and attenuated total reflection infrared (ATR-IR) spectroscopy as well as X-ray photoelectron spectroscopy (XPS).^42,43^ The pyrene-functionalized PNIPAAm attached at both sides and formed a sandwich structure. The thickness of a PNIPAAm monolayer was around 1.4 nm for a number-average molecular weight of ∼10 kDa.^42^ The low film thickness may be explained by the weak π–π-interactions, the large size of the pyrene unit, and the large size of the NIPAAm side chain. The same approach was used by Song et al. to immobilize pyrene-terminated poly(methyl methacrylate)-*b*-polydimethylsiloxane (PMMA-*b*-PDMS) on graphene oxide.^44^ First the PMMA block, and then the PDMS block were grown from a pyrene-based ATRP initiator via ARGET ATRP. The polymer-modified graphene material was then dispersed into pure PMMA to reinforce its mechanical properties and improve the thermal stability and optical properties.

### 2.3 Electrostatic Interactions and Hydrogen Bonds

**Figure 5:**
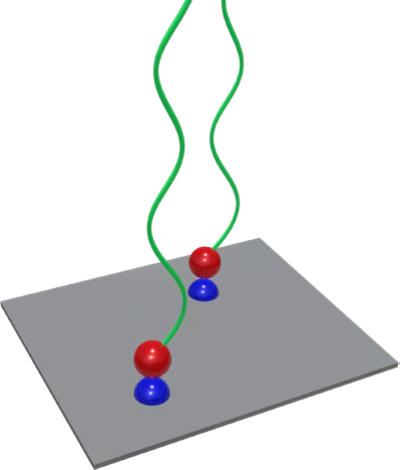
Supramolecular polymer brush tethered via electrostatic interactions.

One of the first studies that noncovalently tethered high molecular weight polymers (>100 kDa) via one functional end-group to a surface using electrostatic interactions (Figure 5) was published in 1988 by Kawaguchi et al.^45^ Polybutadiene with a bis(*p*-diethylamino)phenyl)methanol terminal group could be tethered onto silicon substrates from carbon tetrachloride without any previous modification of the surface. In absence of the bis(*p*-diethylamino)phenyl)methanol end group, the amount of adsorbed polymer was only 50% of the amount of adsorbed end-functionalized polymer. The adsorption was reversible in dioxane and acetone.

Graphene was also functionalized with polymers using electrostatic interactions. Reduced graphene bears carboxylic acid groups on its surface. These were harnessed to immobilize amine end-functionalized PS on the surface. The presence of PS was confirmed with ATR-FTIR as well as Raman spectroscopy. Phase transfer from an aqueous phase into the organic phase, using dichloromethane, *o*-xylene, and benzene occurred upon functionalization with the hydrophobic PS.^46^

**Figure 6:**
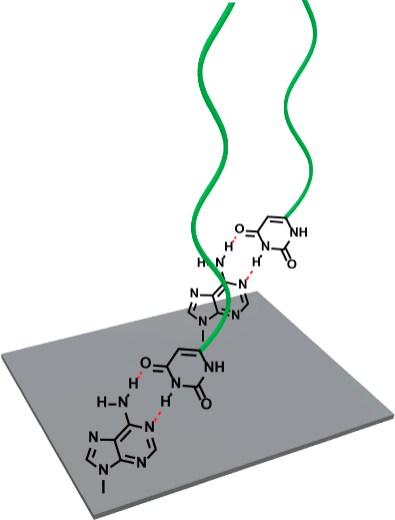
Supramolecular polymer brush tethered via hydrogen bonds.

Another supramolecular motif that has been used for the formation of polymer brushes are (complementary) hydrogen bonds (Figure 6). Viswanathan et al. immobilized an adenine-functionalized triethoxysilane derivative on silica surfaces.^47^ Thymine-terminated PS with a number-average molecular weight (*M*_n_) of 2 kDa was synthesized using anionic polymerization. The polymer was immobilized on the adenine-bearing silicon surface in THF, and the successful immobilization was shown with XPS and water contact angle measurements. The surface-anchored polymers could be detached from the surface by washing with DMSO. In a follow-up study, the dependence of the amount of adsorbed material on the adenine surface concentration was investigated.^48^ An adenine surface concentration of 25% was found to be optimal. The surface grafting density decreased if a higher adenine surface concentration was present on the surface. Reasons for this could be steric hindrance at the interphase, or competing interactions between neighboring adenine units. Hydroxy-terminated PS showed only minor adsorption, proving the superior stability of the adenine–thymine interaction by complementary hydrogen bonds compared to single hydrogen bonds.

Rotello and co-workers used complementary hydrogen bonding interactions between diamidopyridine and thymine to immobilize homopolymers and block copolymers on surfaces.^49^ PS-based diblock copolymers prepared with NMP, with only one block being functionalized with side chain diamidopyridine units, formed polymer brushes after being exposed to thymine-bearing gold substrates in a chloroform solution. Successful adsorption was shown with QCM, XPS, water contact angle measurements, and ellipsometry. For a block copolymer with a total number-average molecular weight of 54 kDa, a film thickness of ∼2 nm was measured. The polymer was removed by rinsing with ethanol and chloroform, and it could be reabsorbed from chloroform. As a follow-up study, Rotello and co-workers published the formation of polymer brushes with a PS homopolymer that was end-functionalized with three diamidopyridine units.^50^ A trivalent complex was formed upon contact of a toluene solution of the functionalized polymer with thymine-functionalized silicon substrates. Removal of the polymer brushes was possible only with THF and successful reabsorption from toluene was shown. A molecular weight of 54 kDa led to the same film thickness of 2 nm that was reported for the diblock copolymer.

### 2.4 Host–Guest Interactions

**Figure 7:**
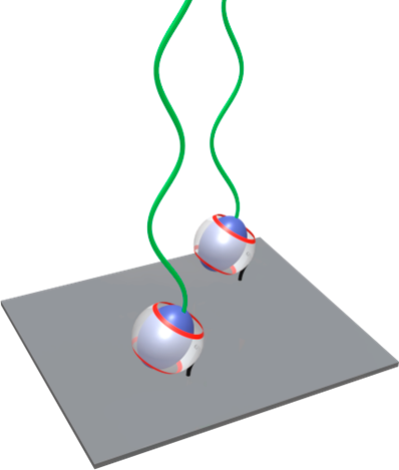
Supramolecular polymer brush tethered via host–guest complexes.

**Figure 8:**
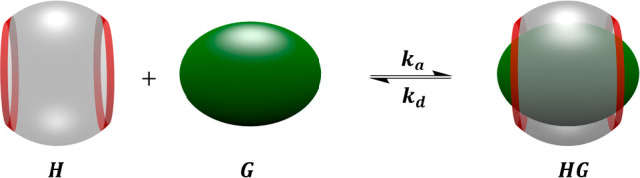
Equilibrium between a host–guest inclusion complex and the free components.^51^

A final class of supramolecular interactions that has been used for the formation of polymer brushes via the “grafting to” approach are host–guest interactions (Figure 7). Host–guest interactions involve the formation of an inclusion complex between a macrocyclic host molecule and a guest molecule. Complex formation is reversible and involves the establishment of an equilibrium between the complex, and the free host and guest (Figure 8).

In aqueous media, one important driving force for complex formation is the “hydrophobic effect,” which involves the release of ordered, high-energy solvent molecules in the host cavity, resulting in favorable entropic and enthalpic changes. However, also other noncovalent interactions such as van der Waals forces, electrostatic interactions, and hydrogen bonds can contribute to the complex formation. The thermodynamic binding constant *K*_*A*_ is the ratio of the association rate constant *k*_*a*_ and the dissociation rate constant *k*_*b*_, which is equal to the concentration ratio of inclusion complex and the free counterparts (eq 3).^51^
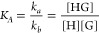
3

**Scheme 1:**
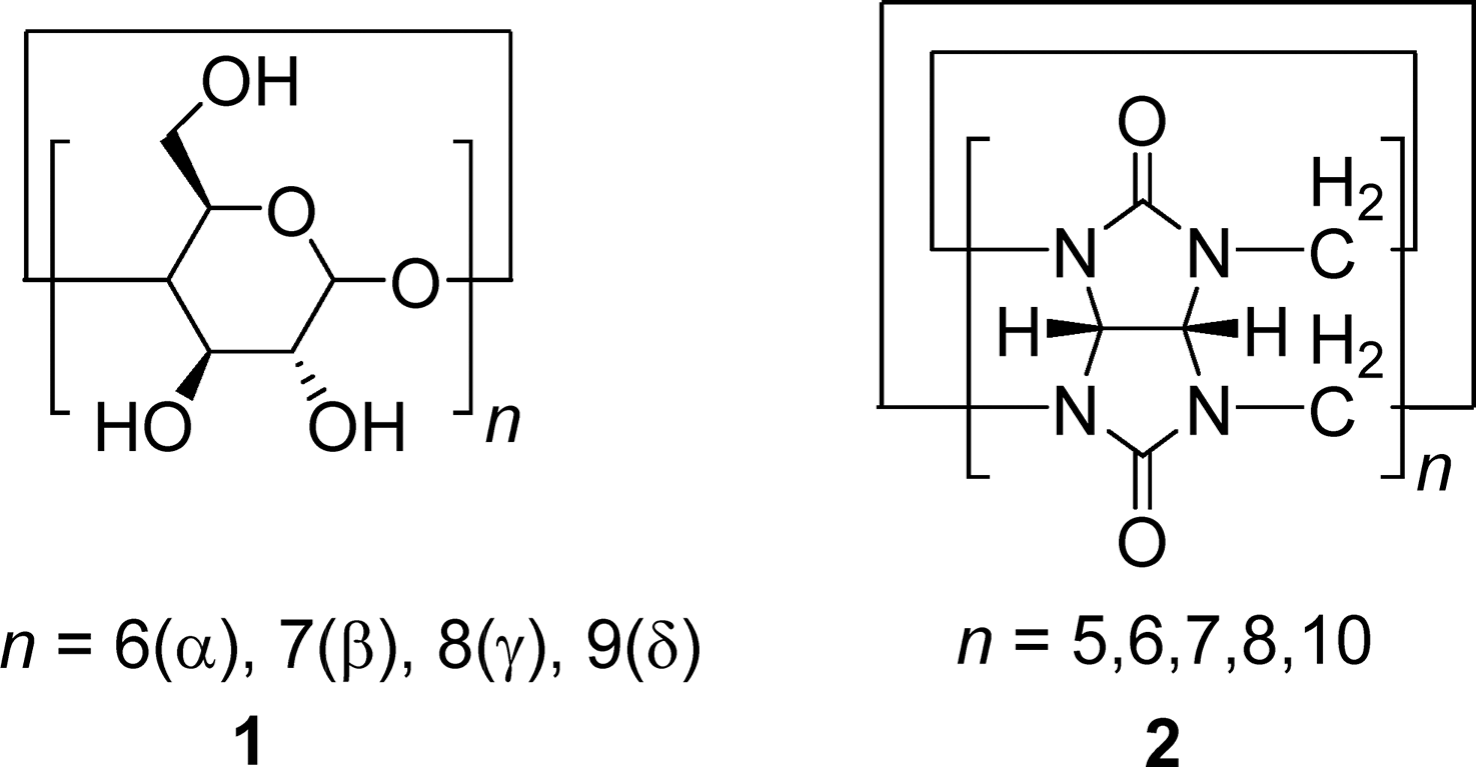
Structure of Cyclodextrins **(1)** and Cucurbit[*n*]urils **(2)**

The rise and development of the field of supramolecular chemistry over the past 40+ years has resulted in the discovery and characterization of a wide range of well-defined host–guest complexes.^52,53^ Among these, as far as we are aware, only cyclodextrin (CD) (**1**) and cucurbit[*n*]uril (CB[*n*]) (**2**) have been used to tether polymer brushes to surfaces. The structure of these macrocycles is shown in Scheme 1. To the family of CDs as well as CB[*n*]s belong several homologues that differ with respect to the numbers of repeating units, and thus ring dimensions. CDs are composed of 6 to 9 α-(1 → 4)-linked glucopyranose units, which are referred to as α-, β-, γ-, and δ- CD, respectively.^54,55^ The homologue with 7 glucopyranose repeats (β-CD), for example, has an outer diameter of 15.4 Å.^56^ CB[*n*]s, on the contrary, are made of methylene bridge-linked glycoluril monomers. Homologues composed of 5, 6, 7, 8, and 10 repeating units (*n* = 5, 6, 7, 8 and 10) have been isolated. CB[7] has an outer diameter of 16.0 Å and shows therefore close dimensional similarity with β-CD.^57^ Both macrocycles are able to bind small organic guests in aqueous solution. However, the binding strengths of the respective complexes are different with CB[7] forming stronger complexes to a variety of guests. Adamantane derivates, for example, are encapsulated in CB[7] with a binding constant of log *K*_*A*_ = ∼ 12, compared to β-cyclodextrin, which forms complexes with binding constants of log *K*_*A*_ = ∼3–4 with the same guest molecules.^54,57^ Recent advances in the (mono)functionalization of these macrocycles enable both the covalent attachment to a substrate or to a polymer chain, and paved the way to host–guest-tethered polymer brushes.^58−60^ Shen et al., for example, prepared UV-switchable surfaces by immobilizing azobenzene-terminated propyl triethoxysilane as the guest component on silica surfaces, followed by grafting of β-CD-terminated PEG, PMMA and poly(methyl methacrylate-*co*-hexafluorobutyl methacrylate) (P(MMA-*co*-HFBMA)).^61^ The polymers were prepared by copper catalyzed azide–alkyne cycloaddition reaction between azido-functionalized β-CD with the alkyne terminated polymers. The formation of the polymer brushes was proven with water contact angle measurements, XPS and AFM. Upon irradiation with UV light, the surface-bound azo-moieties underwent trans-to-cis isomerization, and the polymer chains were released from the surface. Guest surface-attachment was also used by Wang et al. to graft β-CD-tethered poly(2-methacryloyloxyethyl phosphorylcholine) (PMPC) onto silica surfaces.^62^ In this example, not azobenzene, but adamantane guest molecules were covalently immobilized on the substrate. Friction-induced defects in the polymer layer could be repaired simply by exposing the damaged sample to a solution of β-CD-terminated PMPC.

**Figure 9:**
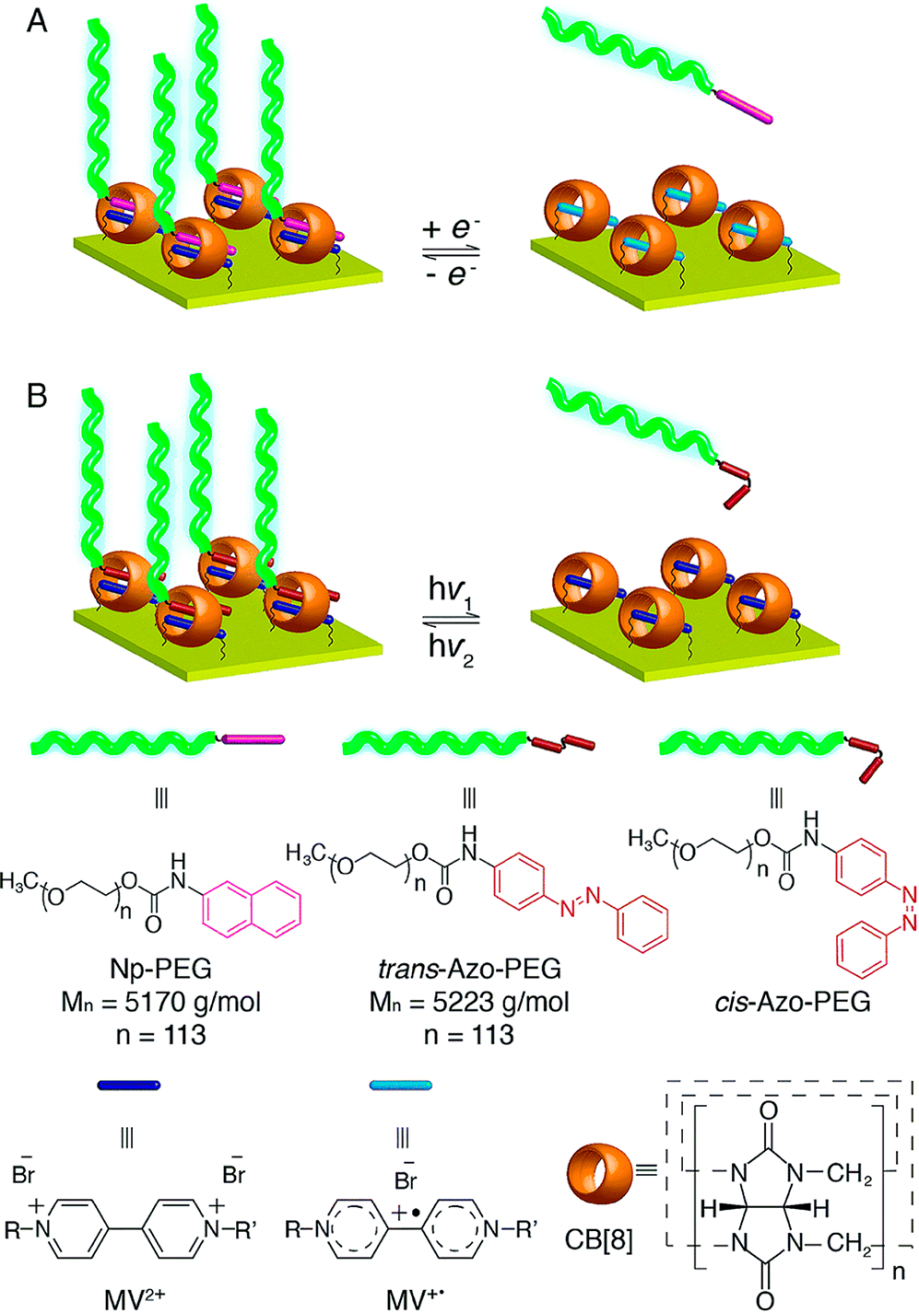
Stimuli-responsive PEG brushes based on CB[8]-based ternary complexes with viologen- and naphthyl- or azobenzene derivates as guest molecules. Stimuli responsiveness can be achieved via (A) reduction of the viologen guest or (B) irradiation-induced trans–cis isomerization of the azobenzene guest.^66^ Reprinted with permission from ref (66). Copyright 2022 Royal Society of Chemistry.

Host–guest-tethered polymer brushes can also be prepared by immobilizing the host molecule covalently on the substrate, and grafting a guest-terminated polymer. Zhao and co-workers used this approach for the preparation of β-CD-tethered polymer brushes. The first step involved the covalent immobilization of amine-functionalized β-CD grafted via an epoxide ring opening reaction onto silicon substrates.^63^ Afterward, azobenzene terminated poly(2-(methacryloyloxy)ethyl)trimethylammonium chloride) (PMTAC), and poly(sodium-4-vinylbenzenesulfonate-*co*-sodium acrylate) (PSS-*co*-AANa) were grafted to the surface from aqueous solution. UV-irradiation dissociated the host–guest complexes and released the polymer brushes. Irradiation with visible light allowed the rebinding of the polymer chains and therefore a reversible switching between both types of polymers. The different bioactivities of the two polymers allowed for the preparation of a material with switchable bioadhesion properties. In another study, the same group immobilized adamantane-terminated PNIPAAm and PMTAC on β-CD-modified silicon surfaces that were prepared using the same epoxide ring opening approach.^64^ The temperature-responsive properties of the PNIPAAm polymer brushes enables temperature-switchable bioadhesion properties of the surfaces. The same group also reported the preparation of polymer brushes on poly(ether sulfone) (PES) membranes by modifying it with β-CD and grafting of adamantane-terminated PMETAC, PEG, and PSS-*co*-AANa.^65^ Scherman and co-workers have used cucurbit[8]uril to tether polymer chains onto gold surfaces.^66^ CB[8] is large enough to simultaneously accommodate two guest molecules in its cavity. The two guests are usually an electron donor–acceptor pair.^60^ In the here described case, the CB[8] molecules were tethered onto gold substrates by the formation of a rotaxane that is tethered via two thiol groups. The center of the rotaxane is formed by a viologen unit as the electron accepting guest which is surrounded by one cucurbit[8]uril molecule (Figure 9). PEG with a molecular weight of 5 kDa, terminated with either a naphthyl- or an azobenzene group as the second, electron-donating guest, could then be tethered onto the surface via the formation of a ternary complex. The polymer brushes had a dry film thickness of 19 nm as determined with ellipsometry. Incubation in water for 2 days decreased the film thickness by only ∼25%. Upon exposure to a poor solvent such as toluene, the polymer chains collapsed on the surface and prevent complex dissociation. However, the polymer brushes could be completely detached from the surface by using a low molecular weight competitive guest, such as naphthol, by reduction of the naphthyl guest, or by irradiation with UV light. Reassembly of the polymer chains was successfully proven with water contact angle measurements and ellipsometry. The same concept was used to prepare micropatterned surfaces with azo benzene- or naphthol terminated PEG_5000_ brushes, by using monolayer colloidal crystal (MCC) templated self-assembly.^67^

## 3 Supramolecular Polymer Brushes Prepared via “Grafting From”

While there are quite a few examples of noncovalently tethered polymer brushes that have been prepared by the “grafting to” method, the “grafting from” method from supramolecular initiators is much less explored. Supramolecular polymer brushes can be prepared via two different “grafting from” approaches, which will be further discussed below. A first approach uses macroinitiators, i.e., polymers, which are physisorbed to the surface of interest and incorporate multiple functional groups that can initiate chain growth. The second approach rests on the use of small molecule initiators that are attached to the surface of interest via noncovalent interactions.

### 3.1 Macroinitiators

**Figure 10:**
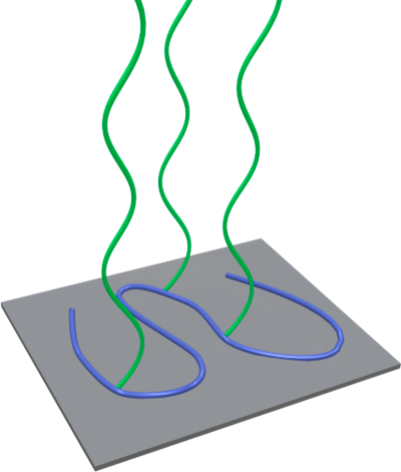
Supramolecular polymer brush grown from macroinitiators.

The use of physisorbed macroinitiators is the most well-established “grafting from” approach for the growth of supramolecular polymer brushes (Figure 10). In this case, a polymer film that contains covalently bound initiator groups is deposited on an underlying substrate and attached via multiple noncovalent bonds. The stability of the adsorbed macroinitiator is due to multivalent and possible cooperative interactions. The increased stability and adhesive strength of molecules tethered via multiple noncovalent bonds has been shown by several studies.^68,69^

One of the first macroinitiators for surface-initiated radical polymerization was developed by Stöhr and Rühe.^70^ The macroinitiator was a monolayer of poly(ε-caprolactone) with covalently bound azo groups that were incorporated in the polymer backbone. These polymers were physically deposited onto silicon substrates from toluene, and they were attached most likely via hydrogen bonds between the silicon oxide surface and the carbonyl oxygens of the macroinitiator. Surface-initiated polymerization from these macroinitiators (which was performed in a nonsolvent for the macroinitiator) results in fragmentation of the macroinitiator backbone, and it generates block copolymer type brushes with a structure that resembles that of a physisorbed block copolymer, although it was grown via the “grafting from” approach. Using this method, polymer brush films with thicknesses of up to 100 nm could be obtained.

Electrostatic interactions have also been harnessed to anchor macroinitiators on silicon substrates. Armes and co-workers developed both cationic as well as anionic polyelectrolyte macroinitiators for the modification of unmodified silica as well as amine-modified silica, respectively. The cationic macroinitiator was prepared by copolymerization of 2-(dimethylamino)ethyl methacrylate (DMAEMA) with 2-hydroxyethyl methacrylate (HEMA), followed by quaternization of the amino group, and reaction of the hydroxyl group with 2-bromoisobutyryl bromide (BiBB). The macroinitiator was deposited on silica from aqueous solution, and a variety of water-soluble monomers could be polymerized via SI-ATRP from spherical^71,72^ and flat^73^ substrates. Anionic macroinitiators contained a mixture of 2-sulfobenzoic acid and isobutyryl bromide groups as anchoring groups and initiator groups, respectively. This macroinitiator was synthesized by postpolymerization modification of PHEMA^74^ or poly(glycerol methacrylate)^75^ with BiBB and 2-sulfobenzoic acid cyclic anhydride. The silica surfaces were premodified with (3-aminopropyl)triethoxysilane before deposition of the copolymer in aqueous solution overnight. The initiator density was higher for the anionic macroinitiator, which resulted in higher ellipsometric film thicknesses after the SI-ATRP of HEMA with 33 nm (anionic) and 12 nm (cationic).

In section 2.2, the grafting of polymer brushes to graphene via π–π-interactions was described. The same binding motif has also been used to immobilize macroinitiators on graphene for the subsequent SI-ATRP. Zhou and co-workers, for example, developed pyrene-containing macroinitiators to grow PNIPAAm, PDMAEMA, poly(3-sulfopropyl methacrylate potassium salt) (PSPMA), and PMTAC brushes from graphene oxide (GO).^76,77^ For the synthesis of the macroinitiator, pyrene, catechol, and bromoisobutyryl side group-containing methacrylates were copolymerized, and the macroinitiator was deposited on the samples via microcontact printing (μCP), followed by the ATRP of the above-mentioned monomers to generate patterned brushes with film thicknesses of up to 100 nm. In a follow-up study, the same process was used to polymerize glycidyl methacrylate (GMA) from GO for the construction of DNA arrays. The film thicknesses of these PGMA brushes were around 110 nm.^78^ Mizutani et al. harnessed π–π-interactions to immobilize a poly(4-vinylbenzyl chloride) macroinitiator onto polystyrene substrates from a DMSO solution. After the deposition of the macroinitiator, NIPAAm was polymerized using ATRP. Due to the temperature-responsive properties of PNIPAAm, a surface with switchable bioadhesive properties toward endothelial cells could be prepared.^79^

### 3.2 Low Molecular Weight Supramolecular Initiators

In contrast to the use of macroinitiators, small molecule initiators anchored via noncovalent interactions have been much less explored for the preparation of polymer brushes via the “grafting-from” approach. Only a handful of examples have been reported that use noncovalent interactions to anchor low molecular weight initiators for the growth of polymer brushes. One of the first studies of such a low-molecular weight initiator was published by Cui et al. in 2012.^80^ Harnessing π–π interactions, a pyrene-terminated RAFT agent was self-assembled onto GO sheets in dioxane, followed by RAFT polymerization of (2-dimethyl aminoethyl) acrylate (DMAEA). Characterization of the polymer brush-modified GO sheets was performed using AFM, transmission electron microscopy (TEM), ATR-IR spectroscopy, and thermogravimetry analysis (TGA). AFM images recorded before and after the polymerization revealed a film thickness of 1.1 nm for an unmodified GO sheet and a film thickness of 4.0 nm for a PDMAEA/GO/PDMAEA sandwich layer. These sandwich structures were formed because the pyrene-initiator can attach from both sides to the GO sheet. Zhou and co-workers prepared a low molecular weight pyrene-terminated ATRP initiator that was self-assembled on GO, and it was used for the SI-ATRP of DMAEMA, NIPAAm, SPMA, and METAC.^77,81^ After immobilization of the initiator on the GO sheet from acetone, a sandwich-like structure with a total thickness of 4.2 nm was formed after the SI-ATRP of DMAEMA. In a follow-up study, the same low-molecular weight initiator was compared with the macroinitiators described in section 2.2.^77^ The same pyrene-based ATRP initiator was immobilized on GO sheets to polymerize 4-vinylphenylboronic acid as reported by Zhang and co-workers.^82^ The modified GO sheets were characterized with FTIR spectroscopy, transmission electron microscopy and XPS. Another low molecular weight supramolecular initiator that harnessed a combination of π–π-interactions and hydrogen bonds was reported by Xiao et al.^83^ Amine-functionalized pyrene (1-pyrenemethylamine) self-assembled on silicon substrates by first, forming a hydrogen bond between an amino group and a hydroxyl group from the silicon surface and second, by forming a π–π-stack with another 1-pyrenemethylamine molecule in ethanol. Afterward, photopolymerization of DMAEMA was initiated by irradiating the modified silicon substrates in a bulk monomer solution with UV light. A film thicknesses of 230 nm was measured with AFM.

**Figure 11:**
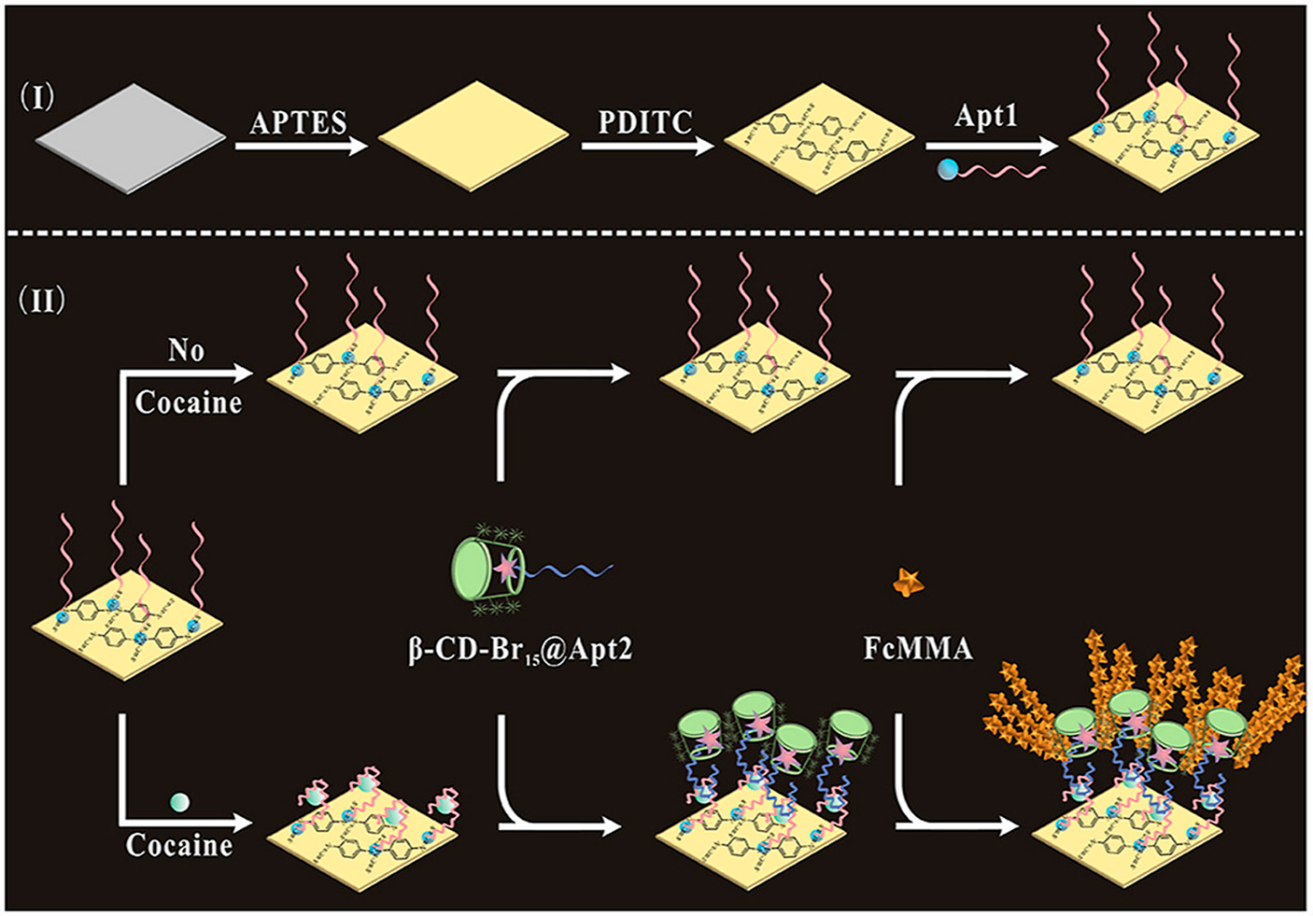
Detection of cocaine with a β-CD/ATRP of FcMMA-based electrochemical approach.^85^ Reprinted with permission from ref (85). Copyright 2022 Elsevier.

Ippel et al. utilized the self-complementary ureido–pyrimidinone (UPy) hydrogen bonding motif for the noncovalent immobilization of initiators for the SI-ATRP of sulfobetaine methacrylate. These polymer brushes were grafted from substrates that were obtained by mixing a UPy-functionalized isobutyryl bromide derivative with a polycaprolactone polymer that was modified with one UPy unit at each chain end. Different ratios of both components allowed control over the initiator density and the morphology of the resulting polymeric material.^84^ Supramolecular polymer brushes have also been used as biosensors. A recent example uses SI-ATRP from supramolecular host–guest based initiators for the electrochemical detection of cocaine.^85^ Wang et al. developed an indium tin oxide (ITO) based sensor that initiates ATRP only upon binding of cocaine to a surface-bound DNA derivate. After binding of the cocaine, a β-CD modified with 15 ATRP initiating bromoisobutyryl units forms a surface-bound inclusion complex with the cocaine and subsequent SI-ATRP of ferrocene methacrylate (FcMMA) enabled the detection of cocaine with electrochemical impedance spectroscopy and cyclic voltammetry (Figure 11). Polymer brushes grafted from initiators that are anchored via coordinative bonds have been prepared by Agergaard et al.,^86^ who reported a catecholato–metal-based noncovalent ATRP initiator via the formation of a complex between two catechol units and Al^3+^ or Fe^3+^ that tethers the ATRP initiator to the surface. Subsequent ATRP enabled the preparation of PMMA brushes, which were released upon electrooxidation of the catechol groups.

## 4 Conclusions

In contrast to the use of covalent interactions to anchor polymer chains via their chain ends to solid surfaces, only comparably limited efforts have been made to explore noncovalent interactions and prepare supramolecular polymer brushes (the only exception being block copolymer physisorption). The examples that have been highlighted in this Perspective, however, illustrate that polymer brushes can also be attached to, or grown from surfaces utilizing noncovalent interactions. The reversible nature of the bonds that tether supramolecular brushes to the surface could impact the dynamics of the surface-anchored polymer chains and provide avenues toward reversible and renewable polymer brush films. Supramolecular polymer brushes thus represent an attractive complement to their well-investigated, covalently tethered counterparts. The various noncovalent approaches highlighted in this Perspective differ with respect to selectivity and binding strength. While many of these supramolecular motifs have been well-characterized in solution studies, an open, and important, fundamental question is whether and how surface-anchoring of these supramolecular motifs impacts binding strength and selectivity. Further open questions are how the binding strength of supramolecular motifs affects the film thickness, grafting density and the properties of supramolecular polymer brushes. A particularly interesting class of noncovalent motifs to anchor polymers to, or graft polymers from surfaces, and to answer some of these fundamental questions, are well-defined host–guest complexes since the binding strengths and selectivity of these motifs can be engineered by molecular design of the host and guest moieties. Further, future research that would address these questions and explore these opportunities would pave the way towards new responsive or renewable polymer brush coatings.

## References

[ref1] Yin L.; Liu L.; Zhang N. Brush-like Polymers: Design, Synthesis and Applications. Chem. Commun. 2021, 57 (81), 10484–10499. 10.1039/D1CC03940G. 34550120

[ref2] Raut A.; Renner P.; Wang R.; Kazadi S.; Mehta S.; Chen Y.; Liang H. Roles of Polymer Brushes in Biological Applications. Adv. Biochips 2021, 2 (1), 12–23. 10.25082/AB.2021.01.001.

[ref3] Zoppe J. O.; Ataman N. C.; Mocny P.; Wang J.; Moraes J.; Klok H. A. Surface-Initiated Controlled Radical Polymerization: State-of-the-Art, Opportunities, and Challenges in Surface and Interface Engineering with Polymer Brushes. Chem. Rev. 2017, 117 (3), 1105–1318. 10.1021/acs.chemrev.6b00314. 28135076

[ref4] Zhao B.; Brittain W. J. Polymer Brushes: Surface-Immobilized Macromolecules. Prog. Polym. Sci. 2000, 25 (5), 677–710. 10.1016/S0079-6700(00)00012-5.

[ref5] Edmondson S.; Osborne V. L.; Huck W. T. S. Polymer Brushes via Surface-Initiated Polymerizations. Chem. Soc. Rev. 2004, 33 (1), 14–22. 10.1039/b210143m. 14737505

[ref6] Azzaroni O. Polymer Brushes Here, There, and Everywhere: Recent Advances in Their Practical Applications and Emerging Opportunities in Multiple Research Fields. J. Polym. Sci. Part A Polym. Chem. 2012, 50 (16), 3225–3258. 10.1002/pola.26119.

[ref7] Barbey R.; Lavanant L.; Paripovic D.; Schüwer N.; Sugnaux C.; Tugulu S.; Klok H.-A. Polymer Brushes via Surface-Initiated Controlled Radical Polymerization: Synthesis, Characterization, Properties, and Applications. Chem. Rev. 2009, 109 (11), 5437–5527. 10.1021/cr900045a. 19845393

[ref8] Brittain W. J.; Minko S. A Structural Definition of Polymer Brushes. J. Polym. Sci. Part A Polym. Chem. 2007, 45 (16), 3505–3512. 10.1002/pola.22180.

[ref9] Galvin C. J.; Genzer J. Applications of Surface-Grafted Macromolecules Derived from Post-Polymerization Modification Reactions. Prog. Polym. Sci. 2012, 37 (7), 871–906. 10.1016/j.progpolymsci.2011.12.001.

[ref10] Ritsema Van Eck G. C.; Chiappisi L.; De Beer S. Fundamentals and Applications of Polymer Brushes in Air. ACS Appl. Polym. Mater. 2022, 4, 3062–3087. 10.1021/acsapm.1c01615. 35601464 PMC9112284

[ref11] Chen W.-L.; Cordero R.; Tran H.; Ober C. K. 50th Anniversary Perspective : Polymer Brushes: Novel Surfaces for Future Materials. Macromolecules 2017, 50 (11), 4089–4113. 10.1021/acs.macromol.7b00450.

[ref12] Moad G.; Rizzardo E.; Thang S. H. Living Radical Polymerization by the RAFT Process – A Second Update. Aust. J. Chem. 2009, 62 (11), 1402–1472. 10.1071/CH09311.

[ref13] Moad G.; Rizzardo E.; Thang S. H. Living Radical Polymerization by the RAFT Process – A Third Update. Aust. J. Chem. 2012, 65 (8), 985 10.1071/CH12295.

[ref14] Lamontagne H. R.; Lessard B. H. Nitroxide-Mediated Polymerization: A Versatile Tool for the Engineering of Next Generation Materials. ACS Appl. Polym. Mater. 2020, 2, 5327–5344. 10.1021/acsapm.0c00888.

[ref15] Brinks M. K.; Studer A. Polymer Brushes by Nitroxide-Mediated Polymerization. Macromol. Rapid Commun. 2009, 30 (13), 1043–1057. 10.1002/marc.200800720. 21706568

[ref16] Nicolas J.; Guillaneuf Y.; Lefay C.; Bertin D.; Gigmes D.; Charleux B. Nitroxide-Mediated Polymerization. Prog. Polym. Sci. 2013, 38 (1), 63–235. 10.1016/j.progpolymsci.2012.06.002.

[ref17] Matyjaszewski K. Advanced Materials by Atom Transfer Radical Polymerization. Adv. Mater. 2018, 30 (23), 1706441 10.1002/adma.201706441. 29582478

[ref18] Pyun J.; Kowalewski T.; Matyjaszewski K. Synthesis of Polymer Brushes Using Atom Transfer Radical Polymerization. Macromol. Rapid Commun. 2003, 24 (18), 1043–1059. 10.1002/marc.200300078.

[ref19] Currie E. P. K.; Norde W.; Cohen Stuart M. A. Tethered Polymer Chains: Surface Chemistry and Their Impact on Colloidal and Surface Properties. Adv. Colloid Interface Sci. 2003, 100–102, 205–265. 10.1016/S0001-8686(02)00061-1. 12668330

[ref20] Ansarifar M. A.; Luckham P. F. Measurement of the Interaction Force Profiles between Block Copolymers of Poly(2-Vinylpyridine)/Poly(t-Butylstyrene) in a Good Solvent. Polymer (Guildf) 1988, 29 (2), 329–335. 10.1016/0032-3861(88)90342-4.

[ref21] Marra J.; Hair M. L. Interactions between Two Adsorbed Layers of Poly(Ethylene Oxide)/Polystyrene Diblock Copolymers in Heptane—Toluene Mixtures. Colloids Surf. 1988, 34 (3), 215–226. 10.1016/0166-6622(88)80100-8.

[ref22] Watanabe H.; Tirrell M. Measurement of Forces in Symmetric and Asymmetric Interactions between Diblock Copolymer Layers Adsorbed on Mica. Macromolecules 1993, 26 (24), 6455–6466. 10.1021/ma00076a023.

[ref23] Taunton H. J.; Toprakcioglu C.; Fetters L. J.; Klein J. Interactions between Surfaces Bearing End-Adsorbed Chains in a Good Solvent. Macromolecules 1990, 23 (2), 571–580. 10.1021/ma00204a033.

[ref24] Norton L. J.; Kramer E. J.; Bates F. S.; Gehlsen M. D.; Jones R. A. L.; Karim A.; Felcher G. P.; Kleb R. Neutron Reflectometry Study of Surface Segregation in an Isotopic Poly(Ethylenepropylene) Blend: Deviation from Mean-Field Theory. Macromolecules 1995, 28 (25), 8621–8628. 10.1021/ma00129a022.

[ref25] Hoogeveen N. G.; Stuart M. A. C.; Fleer G. J. Adsorption of Charged Block Copolymers with Two Adsorbing Blocks. Faraday Discuss. 1994, 98, 161–172. 10.1039/fd9949800161.

[ref26] Hoogeveen N. G.; Cohen Stuart M. A.; Fleer G. J. Can Charged (Block Co)Polymers Act as Stabilisers and Flocculants of Oxides?. Colloids Surfaces A Physicochem. Eng. Asp. 1996, 117 (1–2), 77–88. 10.1016/0927-7757(96)03699-0.

[ref27] Maas J. H.; Cohen Stuart M. A.; Fleer G. J. Thin Block Copolymers Films: Film Formation and Corrugation under an AFM Tip. Thin Solid Films 2000, 358 (1), 234–240. 10.1016/S0040-6090(99)00705-1.

[ref28] Zhao B.; Brittain W. Polymer Brushes: Surface-Immobilized Macromolecules. Prog. Polym. Sci. 2000, 25 (5), 677–710. 10.1016/S0079-6700(00)00012-5.

[ref29] Marques C.; Joanny J. F.; Leibler L. Adsorption of Block Copolymers in Selective Solvents. Macromolecules 1988, 21 (4), 1051–1059. 10.1021/ma00182a035.

[ref30] Sakai K.; Vamvakaki M.; Smith E. G.; Wanless E. J.; Armes S. P.; Biggs S. Adsorption Characteristics of Zwitterionic Diblock Copolymers at the Silica/Aqueous Solution Interface. J. Colloid Interface Sci. 2008, 317 (2), 383–394. 10.1016/j.jcis.2007.09.072. 17935731

[ref31] Munch M. R.; Gast A. P. A Study of Block Copolymer Adsorption Kinetics via Internal Reflection Interferometry. J. Chem. Soc. Faraday Trans. 1990, 86 (9), 1341 10.1039/ft9908601341.

[ref32] Motschmann H.; Stamm M.; Toprakcioglu C. Adsorption Kinetics of Block Copolymers from a Good Solvent: A Two-Stage Process. Macromolecules 1991, 24 (12), 3681–3688. 10.1021/ma00012a032.

[ref33] Guzonas D.; Boils D.; Hair M. L. Surface Force Measurements of Polystyrene-Block-Poly(Ethylene Oxide) Adsorbed from a Nonselective Solvent on Mica. Macromolecules 1991, 24 (11), 3383–3387. 10.1021/ma00011a053.

[ref34] Liu Y.; Quinn J.; Rafailvoich M. H.; Sokolov J.; Zhong X.; Eisenberg A. Neutron Reflectivity Study of Poly(Vinyl-4-Pyridine)-Deuterated Polystyrene (P4VP-DPS) Diblock Brushes. Macromolecules 1995, 28 (18), 6347–6348. 10.1021/ma00122a048.

[ref35] Zhu H.; Masson J. F.; Bazuin C. G. Monolayer Arrays of Nanoparticles on Block Copolymer Brush Films. Langmuir 2019, 35 (15), 5114–5124. 10.1021/acs.langmuir.8b04085. 30905161

[ref36] Parsonage E.; Tirrell M.; Watanabe H.; Nuzzo R. G. Adsorption of Poly(2-Vinylpyridine)-Poly (Styrene) Block Copolymers from Toluene Solutions. Macromolecules 1991, 24 (8), 1987–1995. 10.1021/ma00008a041.

[ref37] Zdyrko B.; Ofir P. B. Y.; Alb A. M.; Reed W. F.; Santore M. M. Adsorption of Copolymers Aggregates: From Kinetics to Adsorbed Layer Structure. J. Colloid Interface Sci. 2008, 322 (2), 365–374. 10.1016/j.jcis.2008.03.047. 18436230

[ref38] Baker S. M.; Smith G. S.; Anastassopoulos D. L.; Toprakcioglu C.; Vradis A. A.; Bucknall D. G. Structure of Polymer Brushes under Shear Flow in a Good Solvent. Macromolecules 2000, 33 (4), 1120–1122. 10.1021/ma991499o.

[ref39] Anastassopoulos D. L.; Spiliopoulos Ν.; Vradis A. A.; Toprakcioglu C.; Baker S. M.; Μenelle Α. Shear-Induced Desorption in Polymer Brushes. Macromolecules 2006, 39 (26), 8901–8904. 10.1021/ma061532o.

[ref40] Mázl Chánová E.; Pop-Georgievski O.; Kumorek M. M.; Janoušková O.; Machová L.; Kubies D.; Rypáček F. Polymer Brushes Based on PLLA- b -PEO Colloids for the Preparation of Protein Resistant PLA Surfaces. Biomater. Sci. 2017, 5 (6), 1130–1143. 10.1039/C7BM00009J. 28498385

[ref41] Layek R. K.; Nandi A. K. A Review on Synthesis and Properties of Polymer Functionalized Graphene. Polymer (Guildf) 2013, 54 (19), 5087–5103. 10.1016/j.polymer.2013.06.027.

[ref42] Liu J.; Yang W.; Tao L.; Li D.; Boyer C.; Davis T. P. Thermosensitive Graphene Nanocomposites Formed Using Pyrene-Terminal Polymers Made by RAFT Polymerization. J. Polym. Sci. Part A Polym. Chem. 2010, 48 (2), 425–433. 10.1002/pola.23802.

[ref43] Jiang D.; Zhu H.; Yang W.; Cui L.; Liu J. One-Side Non-Covalent Modification of CVD Graphene Sheet Using Pyrene-Terminated PNIPAAm Generated via RAFT Polymerization for the Fabrication of Thermo-Responsive Actuators. Sensors Actuators, B Chem. 2017, 239, 193–202. 10.1016/j.snb.2016.08.006.

[ref44] Song S.; Wan C.; Zhang Y. Non-Covalent Functionalization of Graphene Oxide by Pyrene-Block Copolymers for Enhancing Physical Properties of Poly(Methyl Methacrylate). RSC Adv. 2015, 5 (97), 79947–79955. 10.1039/C5RA14967C.

[ref45] Kawaguchi M.; Kawarabayashi M.; Nagata N.; Kato T.; Yoshioka A.; Takahashi A. Adsorption of Polybutadienes with Polar Group Terminations on the Solid Surface. 1. Infrared Study at the Silica Surface. Macromolecules 1988, 21 (4), 1059–1062. 10.1021/ma00182a036.

[ref46] Choi E. Y.; Han T. H.; Hong J.; Kim J. E.; Lee S. H.; Kim H. W.; Kim S. O. Noncovalent Functionalization of Graphene with End-Functional Polymers. J. Mater. Chem. 2010, 20 (10), 1907–1912. 10.1039/b919074k.

[ref47] Viswanathan K.; Ozhalici H.; Elkins C. L.; Heisey C.; Ward T. C.; Long T. E. Multiple Hydrogen Bonding for Reversible Polymer Surface Adhesion. Langmuir 2006, 22 (3), 1099–1105. 10.1021/la052253h. 16430271

[ref48] Viswanathan K.; Long T. E.; Ward T. C. Hydrogen Bonding between Adenine-Modified Surfaces and Terminal Thymine-Functionalized Polystyrene: Influence of the Surface Adenine Concentration on Polymer Recognition. Langmuir 2009, 25 (12), 6808–6812. 10.1021/la803453v. 19459676

[ref49] Sanyal A.; Norsten T. B.; Uzun O.; Rotello V. M. Adsorption/Desorption of Mono- and Diblock Copolymers on Surfaces Using Specific Hydrogen Bonding Interactions. Langmuir 2004, 20 (14), 5958–5964. 10.1021/la049737i. 16459616

[ref50] Xu H.; Norsten T. B.; Uzun O.; Jeoung E.; Rotello V. M. Stimuli Responsive Surfaces through Recognition-Mediated Polymer Modification. Chem. Commun. 2005, (41), 5157–5159. 10.1039/b509572g. 16228020

[ref51] Wagner B. D. Host–Guest Chemistry; De Gruyter: 2020; 10.1515/9783110564389.

[ref52] Dong S.; Zheng B.; Wang F.; Huang F. Supramolecular Polymers Constructed from Macrocycle-Based Host–Guest Molecular Recognition Motifs. Acc. Chem. Res. 2014, 47 (7), 1982–1994. 10.1021/ar5000456. 24684594

[ref53] Murray J.; Kim K.; Ogoshi T.; Yao W.; Gibb B. C. The Aqueous Supramolecular Chemistry of Cucurbit[n]Urils, Pillar[n]Arenes and Deep-Cavity Cavitands. Chem. Soc. Rev. 2017, 46 (9), 2479–2496. 10.1039/C7CS00095B. 28338130 PMC5462124

[ref54] Szejtli J. Introduction and General Overview of Cyclodextrin Chemistry. Chem. Rev. 1998, 98 (5), 1743–1753. 10.1021/cr970022c. 11848947

[ref55] Zhou J.; Ritter H. Cyclodextrin Functionalized Polymers as Drug Delivery Systems. Polymer Chemistry 2010, 1, 1552–1559. 10.1039/c0py00219d.

[ref56] Li Z.; Wang M.; Wang F.; Gu Z.; Du G.; Wu J.; Chen J. γ-Cyclodextrin: A Review on Enzymatic Production and Applications. Appl. Microbiol. Biotechnol. 2007, 77 (2), 245–255. 10.1007/s00253-007-1166-7. 17891389

[ref57] Barrow S. J.; Kasera S.; Rowland M. J.; del Barrio J.; Scherman O. A. Cucurbituril-Based Molecular Recognition. Chem. Rev. 2015, 115 (22), 12320–12406. 10.1021/acs.chemrev.5b00341. 26566008

[ref58] Kasal P.; Jindřich J. Mono-6-Substituted Cyclodextrins—Synthesis and Applications. Molecules 2021, 26 (16), 5065 10.3390/molecules26165065. 34443653 PMC8400779

[ref59] Dong N.; He J.; Li T.; Peralta A.; Avei M. R.; Ma M.; Kaifer A. E. Synthesis and Binding Properties of Monohydroxycucurbit[7]Uril: A Key Derivative for the Functionalization of Cucurbituril Hosts. J. Org. Chem. 2018, 83 (10), 5467–5473. 10.1021/acs.joc.8b00382. 29659280

[ref60] Wiemann M.; Jonkheijm P. Stimuli-Responsive Cucurbit[n]Uril-Mediated Host-Guest Complexes on Surfaces. Isr. J. Chem. 2018, 58 (3–4), 314–325. 10.1002/ijch.201700109.

[ref61] Shen Q.; Liu L.; Zhang W. Fabrication of a Photocontrolled Surface with Switchable Wettability Based on Host–Guest Inclusion Complexation and Protein Resistance. Langmuir 2014, 30 (31), 9361–9369. 10.1021/la500792v. 25053175

[ref62] Wang Y.; Sun Y.; Avestro A.-J.; McGonigal P. R.; Zhang H. Supramolecular Repair of Hydration Lubrication Surfaces. Chem. 2022, 8 (2), 480–493. 10.1016/j.chempr.2021.11.001.

[ref63] Deng J.; Liu X.; Shi W.; Cheng C.; He C.; Zhao C. Light-Triggered Switching of Reversible and Alterable Biofunctionality via β-Cyclodextrin/Azobenzene-Based Host–Guest Interaction. ACS Macro Lett. 2014, 3 (11), 1130–1133. 10.1021/mz500568k. 35610810

[ref64] Shi Z. Q.; Cai Y. T.; Deng J.; Zhao W. F.; Zhao C. S. Host-Guest Self-Assembly Toward Reversible Thermoresponsive Switching for Bacteria Killing and Detachment. ACS Appl. Mater. Interfaces 2016, 8 (36), 23523–23532. 10.1021/acsami.6b07397. 27552087

[ref65] Deng J.; Liu X.; Zhang S.; Cheng C.; Nie C.; Zhao C. Versatile and Rapid Postfunctionalization from Cyclodextrin Modified Host Polymeric Membrane Substrate. Langmuir 2015, 31 (35), 9665–9674. 10.1021/acs.langmuir.5b02038. 26301434

[ref66] Hu C.; Tian F.; Zheng Y.; Tan C. S. Y.; West K. R.; Scherman O. A. Cucurbit[8]Uril Directed Stimuli-Responsive Supramolecular Polymer Brushes for Dynamic Surface Engineering. Chem. Sci. 2015, 6 (9), 5303–5310. 10.1039/C5SC01496D. 28717504 PMC5504464

[ref67] Hu C.; Lan Y.; West K. R.; Scherman O. A. Cucurbit[8]Uril-Regulated Nanopatterning of Binary Polymer Brushes via Colloidal Templating. Adv. Mater. 2015, 27 (48), 7957–7962. 10.1002/adma.201503844. 26509604 PMC4736458

[ref68] Lallemang M.; Yu L.; Cai W.; Rischka K.; Hartwig A.; Haag R.; Hugel T.; Balzer B. N. Multivalent Non-Covalent Interactions Lead to Strongest Polymer Adhesion. Nanoscale 2022, 14 (10), 3768–3776. 10.1039/D1NR08338D. 35171194

[ref69] Gomez-Casado A.; Dam H. H.; Yilmaz M. D.; Florea D.; Jonkheijm P.; Huskens J. Probing Multivalent Interactions in a Synthetic Host–Guest Complex by Dynamic Force Spectroscopy. J. Am. Chem. Soc. 2011, 133 (28), 10849–10857. 10.1021/ja2016125. 21615157

[ref70] Stöhr T.; Rühe J. Monolayers of Amphiphilic Block Copolymers via Physisorbed Macroinitiators. Macromolecules 2000, 33 (12), 4501–4511. 10.1021/ma991193h.

[ref71] Chen X.; Armes S. P. Surface Polymerization of Hydrophilic Methacrylates from Ultrafine Silica Sols in Protic Media at Ambient Temperature: A Novel Approach to Surface Functionalization Using a Polyelectrolytic Macroinitiator. Adv. Mater. 2003, 15 (18), 1558–1562. 10.1002/adma.200305067.

[ref72] Chen X. Y.; Armes S. P.; Greaves S. J.; Watts J. F. Synthesis of Hydrophilic Polymer-Grafted Ultrafine Inorganic Oxide Particles in Protic Media at Ambient Temperature via Atom Transfer Radical Polymerization: Use of an Electrostatically Adsorbed Polyelectrolytic Macroinitiator. Langmuir 2004, 20 (3), 587–595. 10.1021/la0353024. 15773080

[ref73] Chen M.; Briscoe W. H.; Armes S. P.; Cohen H.; Klein J. Robust, Biomimetic Polymer Brush Layers Grown Directly from a Planar Mica Surface. ChemPhysChem 2007, 8 (9), 1303–1306. 10.1002/cphc.200700131. 17510991

[ref74] Vo C. D.; Schmid A.; Armes S. P.; Sakai K.; Biggs S. Surface ATRP of Hydrophilic Monomers from Ultrafine Aqueous Silica Sols Using Anionic Polyelectrolytic Macroinitiators. Langmuir 2007, 23 (2), 408–413. 10.1021/la063003j. 17209588

[ref75] Edmondson S.; Vo C. D.; Armes S. P.; Unali G. F. Surface Polymerization from Planar Surfaces by Atom Transfer Radical Polymerization Using Polyelectrolytic Macroinitiators. Macromolecules 2007, 40 (15), 5271–5278. 10.1021/ma070876r.

[ref76] Wei Q.; Wang X.; Zhou F. A Versatile Macro-Initiator with Dual Functional Anchoring Groups for Surface-Initiated Atom Transfer Radical Polymerization on Various Substrates. Polym. Chem. 2012, 3 (8), 2129–2137. 10.1039/c2py20148h.

[ref77] Gao T.; Wang X.; Yu B.; Wei Q.; Xia Y.; Zhou F. Noncovalent Microcontact Printing for Grafting Patterned Polymer Brushes on Graphene Films. Langmuir 2013, 29 (4), 1054–1060. 10.1021/la304385r. 23294478

[ref78] Gao T.; Ng S. W.; Liu X.; Niu L.; Xie Z.; Guo R.; Chen C.; Zhou X.; Ma J.; Jin W.; Chui Y. S.; Zhang W.; Zhou F.; Zheng Z. Transferable, Transparent and Functional Polymer@graphene 2D Objects. NPG Asia Mater. 2014, 6 (9), e130 10.1038/am.2014.79.

[ref79] Mizutani A.; Kikuchi A.; Yamato M.; Kanazawa H.; Okano T. Preparation of Thermoresponsive Polymer Brush Surfaces and Their Interaction with Cells. Biomaterials 2008, 29 (13), 2073–2081. 10.1016/j.biomaterials.2008.01.004. 18261791

[ref80] Cui L.; Liu J.; Wang R.; Liu Z.; Yang W. A Facile “Graft from” Method to Prepare Molecular-Level Dispersed Graphene-Polymer Composites. J. Polym. Sci. Part A Polym. Chem. 2012, 50 (21), 4423–4432. 10.1002/pola.26264.

[ref81] Gao T.; Ye Q.; Pei X.; Xia Y.; Zhou F. Grafting Polymer Brushes on Graphene Oxide for Controlling Surface Charge States and Templated Synthesis of Metal Nanoparticles. J. Appl. Polym. Sci. 2013, 127 (4), 3074–3083. 10.1002/app.37572.

[ref82] An X.; He X.; Chen L.; Zhang Y. Graphene Oxide-Based Boronate Polymer Brushes via Surface Initiated Atom Transfer Radical Polymerization for the Selective Enrichment of Glycoproteins. J. Mater. Chem. B 2016, 4 (36), 6125–6133. 10.1039/C6TB01489E. 32263501

[ref83] Xiao P.; Gu J.; Chen J.; Han D.; Zhang J.; Cao H.; Xing R.; Han Y.; Wang W.; Chen T. A Microcontact Printing Induced Supramolecular Self-Assembled Photoactive Surface for Patterning Polymer Brushes. Chem. Commun. 2013, 49 (95), 11167 10.1039/c3cc46037a. 24150764

[ref84] Ippel B. D.; Komil M. I.; Bartels P. A. A.; Söntjens S. H. M.; Boonen R. J. E. A.; Smulders M. M. J.; Dankers P. Y. W. Supramolecular Additive-Initiated Controlled Atom Transfer Radical Polymerization of Zwitterionic Polymers on Ureido-Pyrimidinone-Based Biomaterial Surfaces. Macromolecules 2020, 53 (11), 4454–4464. 10.1021/acs.macromol.0c00160. 32581395 PMC7304927

[ref85] Wang J.; Liu J.; Wang M.; Qiu Y.; Kong J.; Zhang X. A Host Guest Interaction Enhanced Polymerization Amplification for Electrochemical Detection of Cocaine. Anal. Chim. Acta 2021, 1184, 339041 10.1016/j.aca.2021.339041. 34625250

[ref86] Agergaard A. H.; Pedersen S. U.; Birkedal H.; Daasbjerg K. Stimuli-Responsive Degrafting of Polymer Brushes: Via Addressable Catecholato-Metal Attachments. Polym. Chem. 2020, 11 (35), 5572–5577. 10.1039/D0PY00916D.

